# The Dynamics of Sensorimotor Cortical Oscillations during the Observation of Hand Movements: An EEG Study

**DOI:** 10.1371/journal.pone.0037534

**Published:** 2012-05-18

**Authors:** Pietro Avanzini, Maddalena Fabbri-Destro, Riccardo Dalla Volta, Elena Daprati, Giacomo Rizzolatti, Gaetano Cantalupo

**Affiliations:** 1 Dipartimento di Neuroscienze – sezione di Fisiologia, Università di Parma, Parma, Italy; 2 Brain Center of Motor and Social Cognition, Italian Institute of Technology, Parma, Italy; 3 Dipartimento di Scienze Mediche e Chirurgiche, Università Magna Graecia, Catanzaro, Italy; 4 Dipartimento di Neuroscienze e Centro di Biomedicina Spaziale, Università Roma Tor Vergata, Roma, Italy; 5 Dipartimento di Neuroscienze – Neuropsichiatria Infantile, Università di Parma, Parma, Italy; The University of Western Ontario, Canada

## Abstract

**Background:**

The observation of action done by others determines a desynchronization of the rhythms recorded from cortical central regions. Here, we examined whether the observation of different types of hand movements (target directed, non-target directed, cyclic and non-cyclic) elicits different EEG cortical temporal patterns.

**Methodology:**

Video-clips of four types of hand movements were shown to right-handed healthy participants. Two were target directed (grasping and pointing) motor acts; two were non-target directed (supinating and clenching) movements. Grasping and supinating were performed once, while pointing and clenching twice (cyclic movements). High-density EEG was recorded and analyzed by means of wavelet transform, subdividing the time course in time bins of 200 ms. The observation of all presented movements produced a desynchronization of alpha and beta rhythms in central and parietal regions. The rhythms desynchronized as soon as the hand movement started, the nadir being reached around 700 ms after movement onset. At the end of the movement, a large power rebound occurred for all bands. Target and non-target directed movements produced an alpha band desynchronization in the central electrodes at the same time, but with a stronger desynchronization and a prolonged rebound for target directed motor acts. Most interestingly, there was a clear correlation between the velocity profile of the observed movements and beta band modulation.

**Significance:**

Our data show that the observation of motor acts determines a modulation of cortical rhythm analogous to that occurring during motor act execution. In particular, the cortical motor system closely follows the velocity of the observed movements. This finding provides strong evidence for the presence in humans of a mechanism (mirror mechanism) mapping action observation on action execution motor programs.

## Introduction

The electrical oscillations recorded from the scalp are typically classified according to their frequency, topography, and reactivity to specific stimuli [1]. The oscillations recorded over sensorimotor regions that desynchronize during active movements are known as mu rhythm.

Mu rhythm was first described under the name of “rolandic rhythm *en arceau*” [2], and considered to belong to the alpha frequency band (8–13 Hz). Subsequent analysis [3], [4] revealed that its arch-like appearance is due to the coexistence of (at least) two not harmonic frequency components whose spectral peaks were distributed around 10 Hz (alpha band) and 20 Hz (beta band). Interestingly, MEG studies [4], [5] hypothesized a spatial segregation between the generators of these two frequency components. This suggestion was based on the observation that the sources of alpha components were identified mainly in somatosensory cortices, whereas the sources of beta components were ascribed primarily to the motor cortex.

Since its first description, it was reported that mu rhythm is blocked by movement execution [2], [6], [7]. A large number of subsequent studies confirmed this observation and quantified the EEG power reduction occurring not only during voluntary movements [8], [9] but also during motor imagery [10].

Although already mentioned by Gastaut [2], the reactivity of mu rhythm to the observation of others' action remained for many years neglected. The discovery of mirror neurons [11]–[13], a set of motor neurons that discharge both during action execution and observation, determined a renewed interest in the cortical motor rhythms not only during action execution, but also during action observation.

A conceptual link between mu rhythm and the mirror neuron activity was first suggested by Altschuler and co-workers [14] and later confirmed by other researchers [15]–[24]. This proposal was based on the reactivity of both mu rhythm and mirror neurons in response to action observation and execution.

The aim of the present study was to explore the correlation between the dynamics of cortical rhythms and some features of the observed movement, analyzing the time-frequency domain with short lasting time-windows. Firstly we characterized the temporal course and topography of somatosensory rhythms during movement observation regardless the action type. Subsequently two issues were addressed in detail. First, whether the observation of target-directed motor acts elicits peculiar EEG activities with respect to the observation of non target-movements; second, whether mu rhythm is modulated by kinematic parameters of the observed movements. Note that the demonstration that the cortical motor rhythms are modulated dynamically during action observation, as it has been previously demonstrated for movement execution [25], would be a crucial evidence for the existence of a mechanism matching the observed action on the analogous motor program in the observer's motor cortex.

## Results

A repeated measure ANOVA with TIME, TARGET, CYCLE, AREA, and HEMISPHERE as within factors was performed for each frequency band. ANOVAs were corrected with the Greenhouse–Geisser (G-Gε) procedure in order to explore the temporal dynamics (see methods section).

The data will be presented as follows. We will describe first the temporal course of the alpha, lower beta and upper beta rhythms power during the observation of movements, regardless of the movement type (main effect: TIME); we will describe then the topography of cortical activities over time (main effects: AREA and HEMISPHERE); we will conclude presenting the relations between specific aspects of the observed movements and cortical rhythms modifications in time and amplitude (main effect: TARGET and CYCLE).

### Temporal course analysis of alpha, lower beta, and upper beta rhythms

**Figure 1:**
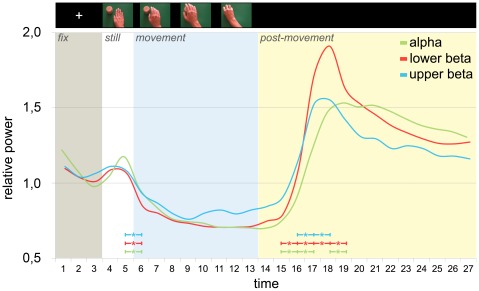
Time Course of EEG Rhythms. The graph shows the EEG power time course for each frequency band: alpha band (8–13 Hz) in green, lower beta (13–18 Hz) in red, and upper beta (18–25 Hz) in cyan. Each epoch (fix, still, movement and post movement) is labeled by a different color. Significant differences between adjacent time bins are indicated by asterisks whose color-code corresponds to that used for different bands. At the top of the figure a film strip shows an example illustrating the different epochs of the observed video clips.

Figure 1 shows the time course of EEG power for alpha, lower beta, and upper beta frequency bands (green, red and cyan curves, respectively) during the observation of all stimuli regardless of type of movements. In all three curves, two early peaks and a later one can be detected. The first two peaks occurred when the fixation cross and the still hand appeared on the screen, respectively. They correspond to visual evoked potentials (VEPs) induced by these phasic events. Note that the second VEP provides evidence that the participants' gaze was directed at the incoming stimuli. The later peak is observed at time bin 18 (3400–3600 ms) for the beta bands and at time bin 19 (3600–3800 ms) for the alpha band.

As soon as the hand movement started, all rhythms reacted showing a desynchronization whose nadir was reached around 700 ms after movement onset (time bin 9). The desynchronization continued during the first 400 ms after the movement offset. The desynchronization was followed by a power rebound [24], [26]. The power rebound amplitude overcomes the baseline value for lower and upper beta band by about 90% and 50%, respectively. The rebound onset of the alpha band was slightly delayed (200 ms) with respect to beta rebound onset.

To exclude that rolandic alpha power reflected a visual areas reactivity volume-conducted to anterior leads, we compared its time course with the occipital one. We observed a strong reduction in occipital alpha power immediately after the fixation cross-induced VEP, while both central and parietal alpha power did not show any significant decrease in the corresponding time bins, thus demonstrating that the central and parietal were not significantly affected by occipital activity. Further evidence that rolandic rhythms were not volume conducted from posterior activities, comes from earlier re-synchronization of occipital alpha rhythm after the end of video-clips, occurring about 400 ms before the centro-parietal one.

In conclusion, temporal analysis revealed that all three rhythms react to movement observation and develop with a similar pattern in time. As far as *alpha-band* (8–13 Hz) is concerned a repeated measures ANOVA revealed a main effect of TIME ([F(26, 260) = 28.31; p<.0001, G-Gε = .13, p<.0001]). Post-hoc comparisons, (p≤.05; Bonferroni-corrected) based on the value of the adjacent time bins revealed significant power differences (transitions) at the beginning of the movement (800–1000 vs 1000–1200 p<.005) and about half a second after the movement end (3000–3200 vs 3200–3400 p<.005; 3200–3400 vs 3400–3600 p<.05). Afterward, alpha-band power slowly decreased to the baseline level.

The same statistical analysis, performed on *lower and upper beta bands*, also showed the main effect of TIME (lower beta [F(26, 260) = 38.20, p<.0001; G-Gε = .10, p<.0001]; upper beta [F(26, 260) = 37.88, p<.0001; G-Gε = .11, p<.0001]). Post-hoc analysis revealed significant power differences (transitions) at the beginning of the movement (800–1000 vs 1000–1200 p<.005 for both bands) and significant rise in both beta bands power about 300 ms after the movement end (2800–3000 vs 3000–3200; lower beta p<.001, upper beta p<.005). This transition towards a hyper-synchronized state continues in the following time bin (3000–3200 vs 3200–3400, lower and upper beta p<.0001), allowing power to reach levels significantly higher than baseline for about one second. The power decrease back to baseline values of lower beta band appears to be steeper with respect to alpha frequency band, resulting in further significant differences (3200–3400 vs 3400–3600 p<.005; 3400–3600 vs 3600–3800 p<.001)

### Topographic Analysis

Both topographic main factors: AREA (central and parietal) and HEMISPHERE (left and right) were found to be not significant. Similarly, no TIMExHEMISPHERE interaction was significant. The interaction AREAxTIME resulted to be significant for upper beta frequency band [F (26, 260) = 6.12, p<.0001; G-Gε = .11, p<.01]. Post-hoc analysis revealed a stronger modulation of parietal with respect to central areas, in particular during the rebound (time bins 17–19, p<.05). Overall, the data suggest a global reactivity common to all fronto-parietal regions.

### Differential effect of type of movements on EEG rhythms

The main factors: TARGET (target-directed and non target-directed movements) and CYCLE (cyclic and non cyclic movements) were found to be not significant

**Figure 2:**
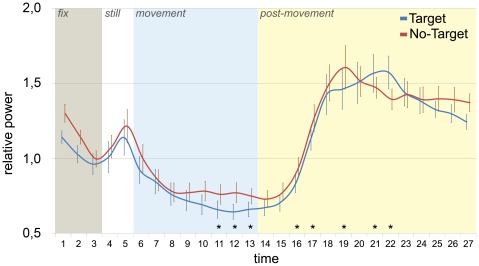
Time course of alpha-band power for target and non-target movements. The graph shows the alpha-band EEG power time course for target (blue line) and non-target (red line) observed movements. Asterisks indicate significant differences between the corresponding bins in the two curves. Each epoch is labeled by a different color.

A significant interaction was found between TIME and TARGET, but only for alpha rhythm [F(26, 260) = 2.45, p<.001; G-Gε = .2, p = .04] (see Figure 2). Post-hoc analysis (p≤.05; Bonferroni-corrected), computed between corresponding time bins of the two conditions (target and non-target), indicated significant differences in both movement and post movement epochs. In particular, target-directed motor acts observation determined a stronger desynchronization than non target-directed ones (time bins 11–13, p = 0.04). Furthermore, it determined a delayed rebound onset (significant time bins 16–17, p = 0.02) and a delayed peak timing for target-directed movements (time bin 19 for non target-directed, p = 0.002; time bin 21–22 for target-directed movements, p<0.01).

**Figure 3:**
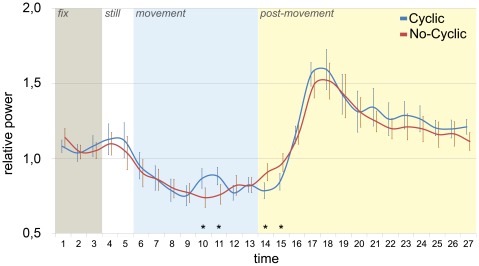
Time course of upper beta rhythm for cyclic and no-cyclic movements. The graph shows the EEG power time course for cyclic (blue line) and non-cyclic (red line) observed movements. Asterisks indicate significant differences between the corresponding bins in the two curves. Each epoch is labeled by a different color.

A significant interaction was found between TIME and CYCLE, but only for lower and upper beta rhythms (lower beta [F(26, 260) = 3.33, p<.0001; G-Gε = .24, p<0.01], upper beta [F(26, 260) = 2.87, p<.0001; G-Gε = .16, p = 0.03]). In particular, post-hoc analysis revealed that, during movement observation, only upper beta band exhibited differences in power profile between cyclic and non cyclic movements, starting about one second after movement onset (Figure 3). More specifically, while the desynchronization of non-cyclic movements (supinating and grasping) showed a monophasic pattern, a transient power increase occurred at time bins 10 and 11 during the observation of cyclic movements (pointing and clenching). Post-hoc comparisons relative to TIME*CYCLE interaction of lower beta band showed significant differences in time bins belonging only to rebound phase (time bin 16–17).

**Figure 4:**
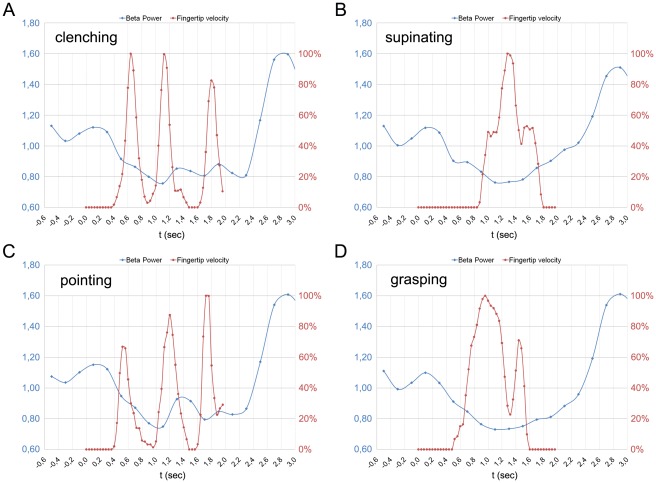
Velocity profile and beta rhythm time course for each single type of movement. The figure includes four panels (A, B, C, and D), one for each observed movements, showing the velocity profiles of observed hand (red line) superimposed on the EEG beta power (blue line). The velocity profiles were computed as the displacement of the fingertip of the actor between subsequent video frames. To make their values comparable, velocity data were normalized to their maximal value.

**Figure 5:**
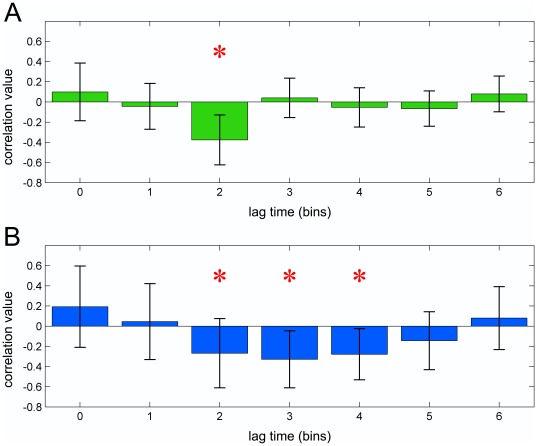
Cross-correlation between the observed velocity and upper beta power. In the figure the mean correlation value and its standard deviation are shown for each considered time lag (only positive values were considered) for both cyclic (panel A, green bars) and non-cyclic (panel B, blue bars) movements. Red asterisks indicate time shifts reaching a significant correlation (p<0.05, Bonferroni corrected).

Figure 4 shows the normalized velocity profiles (red lines) of all four movement types superimposed on the upper beta time course (blue line). This superimposition indicates that two brief power increases consistently occur during cyclic movements (panels A and C). These resynchronizations appear each time hand velocity approximates to zero, with a delay of 400 ms, being the power significantly greater than that of non-cyclic movements only after the end of the first cycle. Furthermore, power values immediately after the end of cyclic movements are significantly lower than those for non-cyclic ones (time bins 14 and 15, Figure 3). Note that cyclic movements in the last 400 ms are still ongoing, while non-cyclic movements are almost terminated. Cross-correlation study between velocity profile and upper beta power showed that cyclic movements exhibited a very strong and significant negative correlation only for a shift of 2 time bins. The same time-shift resulted to be significant even for non-cyclic movements (see Figure 5).

However, due to their regular and monophasic pattern in both velocity and beta power profile, non-cyclic movements showed a negative correlation extended over the values 2, 3 and 4. Considering that, given the same temporal resolution, higher frequency oscillations (like cyclic movement velocity profile) ensure a more focal estimation with respect to slower ones, this finding strongly confirms that velocity profile of the observed movement modulates upper beta power after a delay of 2 time bins, equal to about 400 ms.

The same statistical analysis performed on lower beta rhythm (p≤.05; Bonferroni-corrected) indicated significant differences in time bins belonging only to rebound phase (time bin 16–17).

## Discussion

A large number of brain imaging studies demonstrated that the same parietal and premotor areas active during action execution, are also active during action observation [27]–[29]. Accordingly, TMS studies reported that, during the observation of specific motor acts, an increase of motor evoked potential is observed exclusively in muscles involved in the execution of the same motor act [30], [31]. In line with these findings, EEG and MEG studies showed that desynchronization of central cortical rhythms occurs not only during active movements [2], [5]–[8], but also during action observation [7], [14]–[24], [32]–[34]. These studies focused essentially on a specific alpha-range rhythm (8–13 Hz). However, because of the multispectral nature of mu rhythm [3], [4], in the present study we investigated the reactivity of three sensorimotor frequency bands: alpha (8–13 Hz), lower beta (13–18 Hz) and upper beta (18–25 Hz) bands.

During movement observation our results showed a temporal evolution common to all the considered frequency bands, characterized by a desynchronization, occurring almost as soon as the observed movement starts, and continuing for about 400–600 ms after the movement end. The desynchronization was followed by a prolonged power rebound. The delay in the rebound onset with respect to the movement end possibly corresponds to the time necessary for active inhibition to take place following previous cortical excitation [35]–[36].

Although the similarity in temporal reactivity suggests that the three frequency bands share a common basic mechanism, differences were observed in timing and amplitudes of the rebound in the different frequency bands. The largest rebound was observed in the lower beta band, whose rising phase occurs 200 ms earlier with respect to alpha band rebound. It is important to stress that previous data concerning the execution of hand movements also showed that the lower beta band exhibits the strongest rebound with respect to other frequency ranges [37]. The similarities between these data and the present one are conceptually very interesting because they indicated that cortical dynamics during active movements and action observation share common mechanisms. This is in line with the notion that the mirror mechanism transforms the observed motor act into a motor pattern analogous to that used by the agent.

Previous studies indicated a larger magnitude of reactivity over central areas during movement execution [38]. However, both scalp EEG [39] and subdural ECoG [40] recordings showed almost constantly a spread of the movement-related alpha ERD to the parietal lobe [41], regardless the moved body part. Salmelin et al. [42] reported that alpha and beta maximal reactivity (both ERD and ERS) in movement execution co-localized with the somatosensory evoked fields in post-central regions, suggesting that power rebound was due to sensory afferences. This hypothesis was discarded by the findings that even motor kinesthesic imagery (without actual movement) induces a beta power rebound (see [43]), whose distribution results to be somatotopically organized. Furthermore, Babiloni and coworkers [24], reported a greater alpha band desynchronization over parieto-occipital regions during action observation. The authors ascribed these phenomena to the integration of visual, sensory and motor information occurring in parietal areas.

Our data showed a greater modulation in parietal regions relative to central one, in the upper beta band. This finding may be at first glance surprising if one thinks of the parietal lobe as a mere association areas. However, there is clear evidence since the studies by Mountcastle [44] and Hyvärinen [45] that parietal lobe is a key node in visuomotor transformation both in monkeys [46] and humans [47]. Moreover, recently Arnstein and coworkers demonstrated that, during action observation and execution, mu suppression correlates with a BOLD signal increase in somatosensory cortices (area 2), inferior parietal lobe and dorsal premotor cortex [28].

It is well established by the classical ERD/ERS literature [48] that for voluntary self-paced movements, alpha-band power is reduced over the contralateral hemisphere since 2.5 s before the beginning of the movement. However, this alpha-band ERD becomes bilateral immediately prior to the start of the movement and during the movement execution. Furthermore, even if post-movement beta ERS has been described to be dominant over the contralateral precentral cortex [5], [26], this phenomenon is bilateral [5] and contralateral hemispheric predominance is less consistent after motor imagery [49]. So, in experiments based on action-observation, like the one presented here, there is no pre-movement period and a preparatory ERD is not expected. Thus, it is not surprising that our study confirms findings from most previous studies reporting a bilateral suppression following the observed motion, with no significant difference between hemispheres [19]. Recently, Perry and Bentin [20] showed mu suppression larger in the central electrode contralateral to the observed moving hand. However, the large observation time-period (1 min) analyzed increases the possibility, raised by the authors themselves [20], that participants inadvertently activated hand muscles matching the observed grasp, making it possible that the suppression asymmetry reflected a pre-movement desynchronization. Furthermore, in analogy to what is known for both movement execution [8] and motor/kinesthetic imagery [43], we confirmed previous findings that after each motor event there is a suppression followed by a rebound of alpha and beta power even in action-observation [24]; consequently, pooling data from long observation periods during which movements are repeatedly presented, furnish a measure that is affected by the relative contribution of both suppression and post-movement rebound on alpha and beta power. This latter consideration could also be responsible for most of the conflicting results on lateralization since now discussed.

The comparison between observation of target and non target-directed motor acts showed a greater desynchronization for target-directed movements relative to non target-directed ones. Previous studies also reported significantly lower power values for alpha band during observation of a precision grip (target-directed movement) relative to those during observation of a simple hand extension (non target-direct movement) [19]. Our data also show that this difference appears in the last part of the target-directed movements relative to non target-directed ones, while no difference is present at the very beginning of the movement.

After the movement end, a time shift occurs between the EEG alpha activity relative to the two movement types, culminating in a strongly delayed timing of rebound peak for target-directed with respect to non target-directed movements.

There are two possible explanation of this behavior. The first, more fascinating, is that two different neuronal populations are recruited, according to the presence or not of a target, and that their activation induces a different level of desynchronization and different timing for returning back to a resting state. An alternative explanation is that the same neuronal populations code for both type of movements, but the presence of a target requires more complex processing, leading to a greater desynchronization and a delayed recovery to the baseline level.

Another interesting finding of our study concerns the relations between the kinematics of the observed movements and the upper beta band activity during the desynchronization phase. In particular, while non-cyclic movements determined a monophasic desynchronization curve, cyclic ones elicited a biphasic pattern, with a transient power increase lasting about 400 ms. Furthermore, the post-desynchronization rebound onset for cyclic movements preceded that of the non-cyclic ones of about 400 ms.

Recent observations reported that the velocity of both executed and imagined movement modulates beta frequency band [25], [50], [51]. Furthermore, Press and co-workers [22] reported a similar modulation during the observation of a biological motion of the arm, with the upper beta band power significantly lower 200–250 ms before a midpoint relative to an endpoint.

Here we show the entire time course of upper beta rhythm during movement observation comparing motor acts with different kinematics (cyclic vs. non-cyclic movements). As shown in Figure 4 and 5, a strong correlation was found between the velocity profile of each single movement and the beta power. Indeed, a transient rise in power occurred 400 ms after each time the velocity of the hand approximates to zero. The observation that, during these brief resynchronization periods, the power did not reach the levels of the post-movement rebound, is probably due to the contrasting effect of a rapid desynchronization induced by the overcoming velocity resumption of the movement. This is in line with the movement related desynchronization observed in the first 200 ms after the movement onset (see Results).

It is important to note that these data show a fundamental difference between human and monkey mirror mechanism. The latter codes the goal of the motor acts, but it does not appear to be sensitive to the kinematics of the observed motor acts (for a review see [52]). This is not the case for the human mirror mechanism that, as shown in the present study, also responds to the velocity profile of single movements.

## Materials and Methods

### Participants

Thirteen volunteers (5 female, 8 male; mean age = 25.8±5.5 yrs) took part in the study. All of them were right-handed, as resulting from Edinburgh Inventory [53] (mean score = 0.85±0.1), and had normal or corrected-to-normal vision. The experimental protocol was in line with the Declaration of Helsinki and approved by the local ethics committee (COMITATO ETICO UNICO PER LA PROVINCIA DI PARMA). Before the experiment the participants gave written informed consent for the study.

### Stimuli

**Figure 6:**
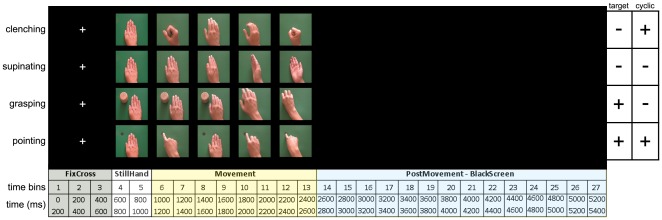
Presented stimuli and timeline of the experiment. In the figure the structure of the administered trial is shown. The upper part shows still frames from the video clips displaying the four different observed movements. Target and cyclic attributes of movements are showed in the rightmost part of the upper panel. In the first line of the lower part, the four epochs of each trial are indicated. The second and third lines show the time bins considered in the analysis and the corresponding time interval (each 200 ms long), respectively.

The stimuli consisted of 4 video clips (each 2000 ms long, 800×640 pixels of resolution) depicting *four* different types of hand movements performed with the right hand (Fig. 6). All stimuli were presented on a dark background with a 19″ LCD monitor (screen resolution 1280×960 pixels). The first 400 ms of all videos showed the hand in a resting position along the midline (STILL epoch). Still hand presentation enabled us to differentiate electrical activity evoked by stimulus occurrence (the hand presentation triggered evoked potentials) from EEG reactivity to movement onset.

The four types of hand movements can be subdivided into two major categories according to the presence either of a target (TARGET-DIRECTED or NON TARGET-DIRECTED) or movement repetition (CYCLIC and NON-CYCLIC) in a 2×2 design. Of the two target-directed movements, one was not cyclic, the other cyclic. The first consisted in a reaching to grasp movement (Grasping, TARGET-DIRECTED, NON-CYCLIC), the second consisted of a pointing movement directed toward a black dot painted on the table, followed by a movement bringing the hand back to its initial position and again to the target (Pointing, TARGET-DIRECTED, CYCLIC). The movements followed an identical trajectory, with the resting hand lying along the observers' midline.

Of the two non-target directed movements one was non-cyclic, the other cyclic. The first consisted of hand supination, i.e. supinating from a palm-down starting position to a palm-up final position (Supinating, NON TARGET-DIRECTED, NON-CYCLIC). The second consisted of hand clenching (starting from the open hand, lifting and closing it) repeated twice (Clenching, NON TARGET-DIRECTED, CYCLIC).

For each video-clip, the velocity profile was computed as the displacement of the fingertip of the actor between subsequent video frames. To make their values comparable, velocity data were normalized to their maximum.

### Procedure

All participants sat on a comfortable armchair at about 60 cm from the monitor. They were instructed to observe the video-clips. To ensure participants' attention, they were asked to tell the color of an X randomly appearing in the middle of the screen one second after the movement onset. The ‘X’ appeared 12 times during an experimental session, 3 times for each action type, in a randomized order. All participants responded with the 100% accuracy. These trials were removed from subsequent analysis. All visual stimuli were administered using E-Prime software (Psychology Software Tools. Inc).

Each trial started with the presentation of a central fixation cross (FIX epoch, 600 ms long) inviting participants to be ready for the subsequent stimuli. A videoclip was then presented (duration 2000 ms) showing a resting hand (STILL epoch, 400 ms) subsequently performing one of the four movements. All video-clips had the same duration (movement epoch, 1600 ms). Also the time period after the end of movement was considered for data analysis. In this post-movement period (random duration between 4 and 8 seconds) the participants saw a dark background, but only the first 2800 ms of post movement were used in the analysis (Post movement, 2800 ms). Summarizing, each trial included four epochs (FIX, STILL, movement, and Post movement). Each stimulus was presented 33 times (30 valid +3 catch trial) for a total amount of 132 stimuli (about 20 minutes long recording). The trial sequence was fully randomized for each participant.

### EEG Acquisition

Continuous EEG was acquired using the 128-channel Geodesic EEG System (Electrical Geodesics, Inc., Eugene, OR, USA) and the HydroCel Geodesic Sensor Net that arrays the sensors (AgCl coated electrodes) in a geodesic pattern over the surface of the head. It included 19 contacts at the equivalent 10–20 system locations. Consistent positioning was achieved by aligning the Sensor Net with skull landmarks (nasion, vertex, and preauricular points). With high input impedance amplifiers (Net Amps300), low noise EEG was obtained with sensor-skin impedances maintained below 100 kΩ. The signal was digitized at 250 Hz sampling rate (0.01 Hz high-pass filter), recorded with a vertex reference.

### EEG Analysis

EEG data were analyzed off-line by means of NetStation software (Electrical Geodesics, Inc., Eugene, OR, USA) and homemade MATLAB scripts (The Mathworks, Natick, MA). Continuous recordings were filtered (band-pass 1–35 Hz) and segmented in epochs lasting 5800 ms, each including baseline (400 ms just preceding fixation), fixation (600 ms, FIX), still hand observation (400 ms, STILL), movement observation (1600 ms, movement) and post movement (2800 ms, Post movement), obtaining an epoch-file containing single-trial data for each subject.

For the artifact detection and removal, each participant's epoch-file was imported in EEGLAB tool [54] and analyzed by means of Independent Component Analysis (ICA) then back-transformed excluding components endowing eye (blink and movement), cardiac, and muscular artifacts. The resulting epoch-files were further visually inspected to exclude remaining “bad trials” (about 6% of trials removed), re-referenced using the average signal of every scalp electrode as reference (excluding those below the axial plane passing through fronto-polar and occipital electrodes), and baseline-corrected. Two subjects were excluded from subsequent analysis because the number of removed trials exceeded 30% of overall trials. For each condition and for each electrode, relative power values were computed by means of a continuous Morlet wavelet transform of single-trial data for the frequency range from 5 to 30 Hz. Afterward, an average panel was computed for each participant and condition.

### Statistical Analysis

Three different frequency bands were considered for data analysis: alpha (8–13 Hz), lower beta (13–18 Hz) and upper beta (18–25 Hz). They were selected on the basis of raw data (see Figure S1) and according to previous studies addressing similar topics [55]–[57]. Previous studies reported modulation of the peak beta frequency across different tasks or epochs in the same task [58]–[59]. We excluded the existence of systematic differences in the peak of the selected frequency bands between conditions by means of preliminary analysis (see Methods S1 and Figure S2).

**Figure 7:**
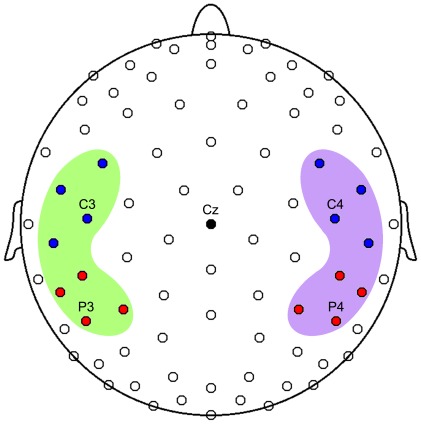
128-channel EEG array. Top view of the scalp where the blue circles indicate electrodes located in the frontal clusters, red ones in parietal clusters. Green area includes left hemisphere electrodes, purple area the right hemisphere ones. The clusters includes C3, C4, P3, P4. In the figure electrodes belonging to occipital clusters (including O1 and O2) are not visible.

The mean power was computed for each 27 consecutive time bins, 200 ms long, for all bands (Figure 6). This time-window duration was chosen taking into account that the period of a single 8 Hz-oscillation is 125 ms; thus for each time-bin the power of at least one complete period was computed. Because we were interested in assessing the responsiveness of the cortical areas included in the observation/execution network (mirror network), two symmetric peri-rolandic clusters of electrodes (one for each hemisphere) were analyzed. They were further subdivided into a central and a parietal set (AREA) according to 10–20 system nomenclature (Figure 7). A separate preliminary analysis including two occipital clusters was also conducted to exclude a possible influence of volume conduction from visual cortices on rolandic alpha component [60].

In order to explore the relationship between EEG power and kinematics of the observed movements, we performed a cross-correlation analysis for each condition and subject. Velocity profiles were downsampled so as to fit with the upper beta power time resolution (obtaining time-series 10-points long), and both curves were normalized between 0 and 1. Subsequently, the cross correlation (Matlab *crosscorr* function) between the two curves was calculated, investigating a shift ranging from 0 (i.e. simultaneous signals) up to 5 time bins (i.e. delay of about 1 second between the two signals). By means of this analysis, we described how the correlation changed introducing different delays between the two curves. No negative shifts were explored, so as to take into account only causal relationship of observed velocity on EEG power.

To assess which factors supply a significant interaction with TIME, a repeated measure ANOVA for each frequency band was performed, with TARGET, CYCLE, AREA, HEMISPHERE and TIME as within factors. ANOVAs were corrected with the Greenhouse–Geisser (G-Gε) [61] procedure in order to explore the temporal dynamics. Post hoc analysis on TIME main effect was performed with planned comparisons design between adjacent time bins, and p-values were calculated with Bonferroni correction.

To investigate the effect of topography (AREA and HEMISPHERE) and movement features (TARGET and CYCLE), post-hoc analyses on interactions of each factor with TIME were performed. Power values across the different levels of each condition were compared into each time bin following a planned comparisons design. Greenhouse-Geisser correction was applied.

## Supporting Information

Figure S1 **TF panel.** Example of TF panel obtained over centro-parietal area for one subject and one condition (grasping). The red dashed horizontal lines show the borders between the selected frequency bands.
(TIF)

Methods S1 Methods employed to validate the frequency bands of interest and to exclude within-subjects systematic differences across conditions.
(DOC)

Figure S2 **Between-subjects peak frequency difference.** Graphs describing the alpha- and beta-peak frequency distributions over all subjects. In the left panels, mean and standard deviation for maximal ERD frequency are reported for both alpha (red line) and upper beta (blue line) band. ANOVA on these values resulted in no significant differences (see p-values on the top of the figure). In the right panels, the curves for each single subject are reported.
(TIF)
