# Supplementary figures and images for: The Dynamics of Sensorimotor Cortical Oscillations during the Observation of Hand Movements: An EEG Study
Source: PLoS One. 2012 May 18;7(5):e37534. doi: 10.1371/journal.pone.0037534 (PMC3356327; doi:10.1371/journal.pone.0037534)

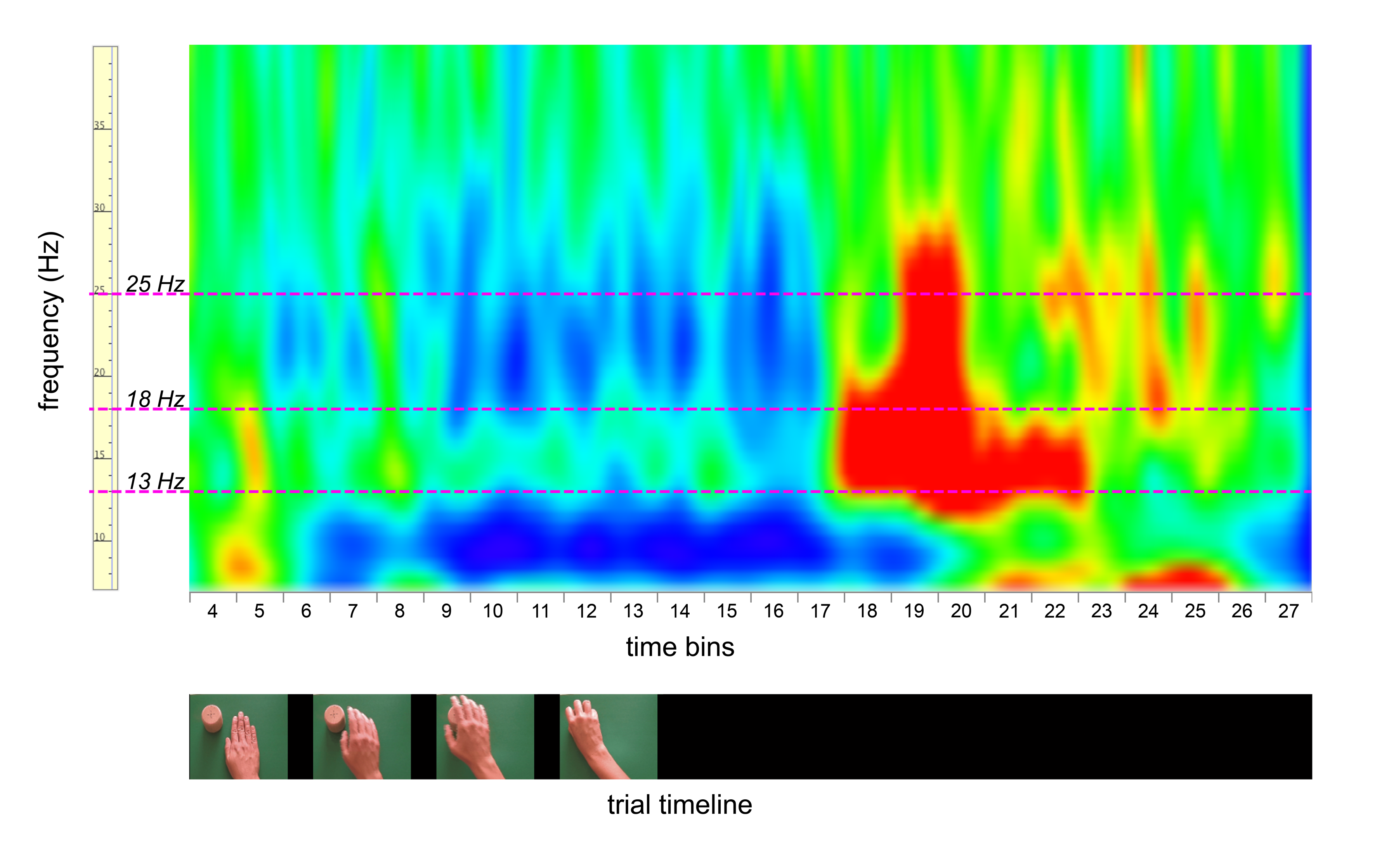

Supplement: Figure S1 — TF panel. Example of TF panel obtained over centro-parietal area for one subject and one condition (grasping). The red dashed horizontal lines show the borders between the selected frequency bands. (TIF) [file pone.0037534.s001.tif]

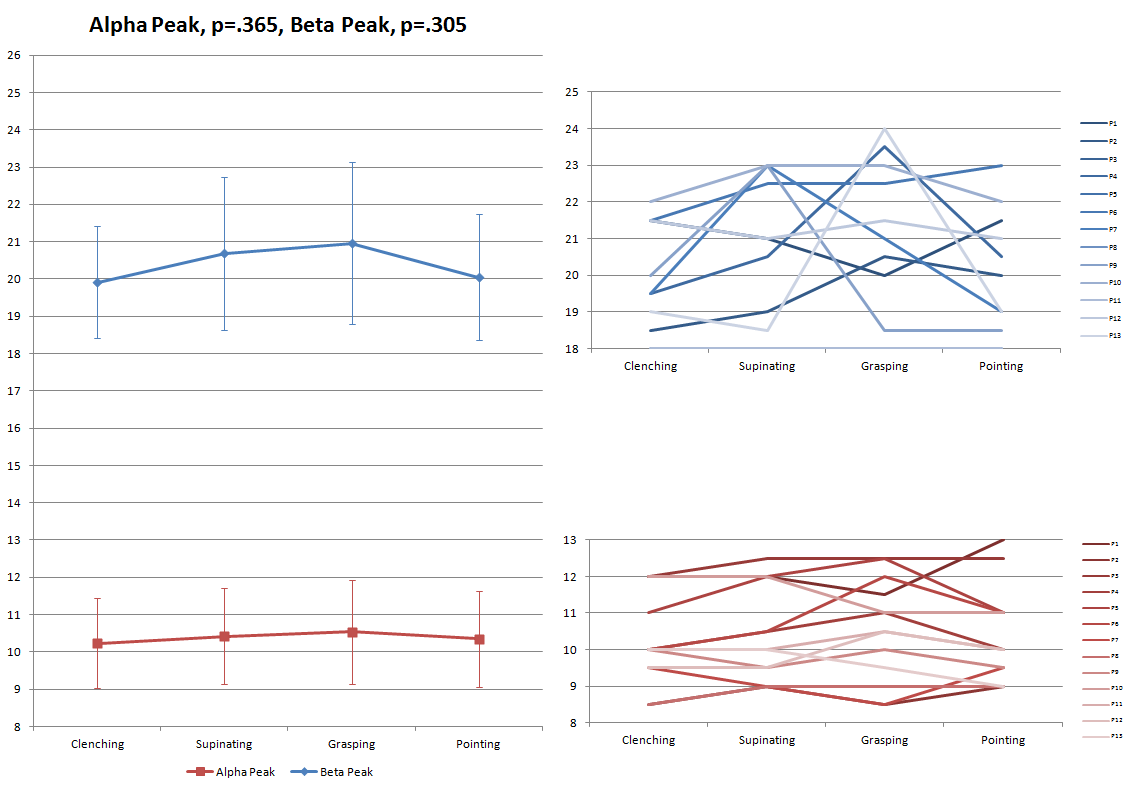

Supplement: Figure S2 — Between-subjects peak frequency difference. Graphs describing the alpha- and beta-peak frequency distributions over all subjects. In the left panels, mean and standard deviation for maximal ERD frequency are reported for both alpha (red line) and upper beta (blue line) band. ANOVA on these values resulted in no significant differences (see p-values on the top of the figure). In the right panels, the curves for each single subject are reported. (TIF) [file pone.0037534.s003.tif]
