# Supplementary material for: The Dynamics of Sensorimotor Cortical Oscillations during the Observation of Hand Movements: An EEG Study
Source: PLoS One. 2012 May 18;7(5):e37534. doi: 10.1371/journal.pone.0037534 (PMC3356327; doi:10.1371/journal.pone.0037534)
Supplement: Methods S1 — Methods employed to validate the frequency bands of interest and to exclude within-subjects systematic differences across conditions. (DOC) [file pone.0037534.s002.doc]

**Supporting Methods S1**

To identify the frequency bands of interest, we assessed the frequency desynchronization peak in all subjects and all conditions. More specifically, we considered all wavelet panels resulting from the time-frequency analysis, subdivided the frequency axis in adjacent bands of 0.5 Hz and, finally, averaged the corresponding power during the whole movement observation epoch. In figure R14a an example of such panel is shown. For each participant and each condition, the alpha- and beta-peak frequency were computed as the bands exhibiting the lowest mean power in their range [8-13 Hz] and [13-30 Hz] during movement observation. Results of this comparison are shown in Figure S1. In the left panel, both alpha (red line) and beta (blue line) peak mean frequencies are reported for each condition separately. Standard deviations are indicated with thin error bars of the same color. We performed a repeated measurement ANOVA on these frequency values, separately for the two bands, finding no significant difference among conditions. Moreover, to describe even the within subject behavior, we report two more graphs: the first one (Figure S2, lower right panel) shows the alpha-peak frequency for all subjects and condition, while the second illustrates the same data relative to beta band. Note that, even if some significant differences exist in the same subject among conditions, no difference resulted to be consistent among subjects.
